# Supplementary material for: Light-based modulation of astrocytic calcium for regulation of organelle dynamics and morphogenesis
Source: J Cell Biol. 2026 May 19;225(7):e202506032. doi: 10.1083/jcb.202506032 (PMC13186144; doi:10.1083/jcb.202506032)
Supplement: Table S1 — shows primers of plasmids. [file jcb_202506032_tables1.docx]

Table S1. Primers of plasmids

| Primers | Sequence (5'→3') |
| --- | --- |
| KIF5C 1-559-mCherry-FKBP-F1-F | TGAACCGTCAGATCCGCTAGCGCCGCCATGGCAGATCCAGCCGAATG |
| KIF5C 1-559-mCherry-FKBP-F1-R | GCCCTTGCTCACCATAAGCTTGGTAGGCCTAGGCGCGCC |
| KIF5C 1-559-mCherry-FKBP-F2-F | ATGGTGAGCAAGGGCGAGGAG |
| KIF5C 1-559-mCherry-FKBP-F2-R | TCTAGAGACGGTCCGACTAGTCTTGTAC |
| KIF5C 1-559-mCherry-FKBP-F3-F | CGGACCGTCTCTAGAGGAGTGCAGGTGGAAACCATC |
| KIF5C 1-559-mCherry-FKBP-F3-R | CGGGCCCGCGGTACCGTCGACTTATTCCAGTTTTAGAAGCTCCACATCG |
| mCherry-BicD2 1-594-FKBP-F | ACGCGTCGACTCTCATTCCGCGCCGTC |
| mCherry-BicD2 1-594-FKBP-R | CGCGGATCCTTCCAGTTTTAGAAGCTCCACATC |
| myc-FRB-Halo-F1-F | TCCCCCGGGCTGCAGGAATTCATCCTCTGGCATGAGATGTGG |
| myc-FRB-Halo-F2-R | TCCGTACGGATAACTAGTCTTTGAGATTCGTCGGAACAC |
| myc-FRB-Halo-F3-F | ACTAGTTATCCGTACGGAATGGCAGAAATCGGTACTGGC |
| myc-FRB-Halo-F4-R | GGGCCCCCCCTCGAGAAGCTTGCCGGAAATCTCGAGCGTC |
| mCherry-Rab 5a-F | GCTTCGAATTCTGCA ATGGCTAATCGAGGAGCAAC |
| mCherry-Rab 5a-R | TTATCTAGATCCGGT GTTACTACAACACTGACTCCTGG |
| mCherry-Rab 11a-F | AGTCCGGACTCAGATCTCGAGGTATGGGCACCCGCGACG |
| mCherry-Rab 11a-R | GTACCGTCGACTGCAGAATTCTTAGATGTTCTGACAGCACTG |
| Myc-Glut4-FRB-Halo-F | GATCTGAGCCCGGGCGGATCC ATGCCGTCGGGTTTCCAGC |
| Myc-Glut4-FRB-Halo-R | CTCATGCCAGAGGAT GAATTCGACCGGTGGATCCCCGTCATTC |
| Glut4-mCherry-F | CTACCGGACTCAGATCTCGAGACAAGATGCCGTCGGGTTTCCAGCAG |
| Glut4-mCherry-R | GGTGGCGACCGGTGGATCCCCGTCATTCTCATCTGGCCCTAAGTATTC |
| myc-PEX3 1-43 aa-FRB-Halo-F | TCAGATCTCGAGCTCGCCGCCATGCTGAGGTCTGTATGGAATTTTCTG |
| myc-PEX3 1-43 aa-FRB-Halo-R | CTCATGCCAGAGGATGAATTCCTGCAGCCCGGGCTCCCTTTCCTG |
| PEX3 1-43 aa-mCherry-F | TCAGATCTCGAGCTCGCCGCCATGCTGAGGTCTGTATGGAATTTTCTG |
| PEX3 1-43 aa-mCherry-R | ATGGTGGCGACCGGTGAGAATTCCTGCAGCCCGGGC |
| Myc-FRB-halo-MoA 490-527-F | CTCGAGATTTCCGGCAGTGCTGGTGGTAGTGC |
| Myc-FRB-halo-MoA 490-527-R | GGGCCCCCCCTCGAGTCAAGACCGTGGCAGGAG |
| Myc-LAMP1 1-417 FRB-halo | GATCTGAGCCCGGGCTCGAGCGCCACCATGGC |
| LAMP1-mCherry-F | ACTCAGATCTCGAGCGCCACCATGGCGGCCCCGGGC |
| LAMP1-mCherry-R | CCGGTGGAGCCGAATTCCGAGATGGTCTGATAGCCCGCGTG |
