# Supplementary material for: Light-based modulation of astrocytic calcium for regulation of organelle dynamics and morphogenesis
Source: J Cell Biol. 2026 May 19;225(7):e202506032. doi: 10.1083/jcb.202506032 (PMC13186144; doi:10.1083/jcb.202506032)

Fig. 2D

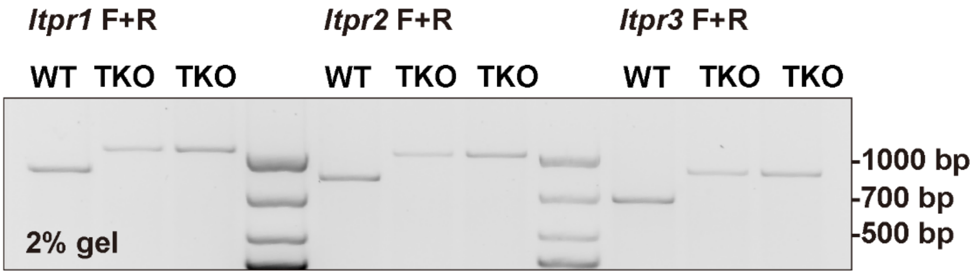
Itpr1 F+R
Itpr2 F+R
Itpr3 F+R
WT TKO TKO
WT TKO TKO
WT TKO TKO
2% gel
1000 bp
700 bp
500 bp

Source data for Fig. 2D **Original image file from which Fg. 2D was assembled.**

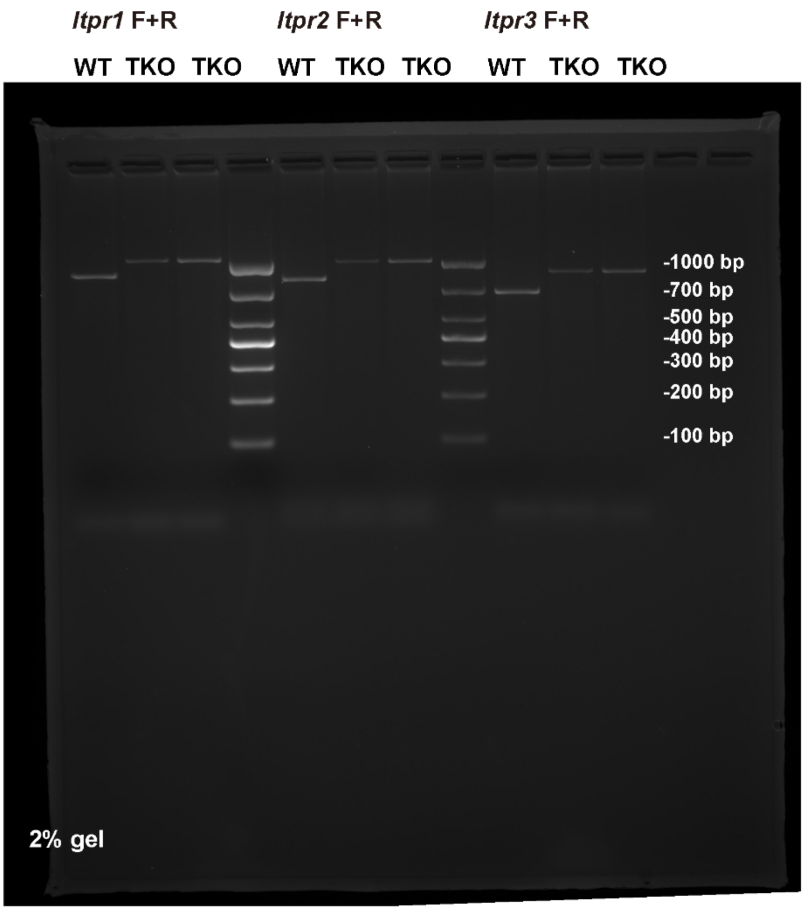

Primers for genotyping

Itpr1	F: AGACCTCTGCCTTAGGAGGTATTT<br>R: TTTAAGAAAGCAAGGAGAAGGAGA
Itpr2	F: GCTGTGCCCAAAATCCTAGCACTG<br>R: AGTGATACAGGGCAAGTTCATAC
Itpr3	F: CCTGCCTCCGTTTGTTACAT<br>R: AGCTCCAGGTCTATAAAGCAAATG

Fig. 2E

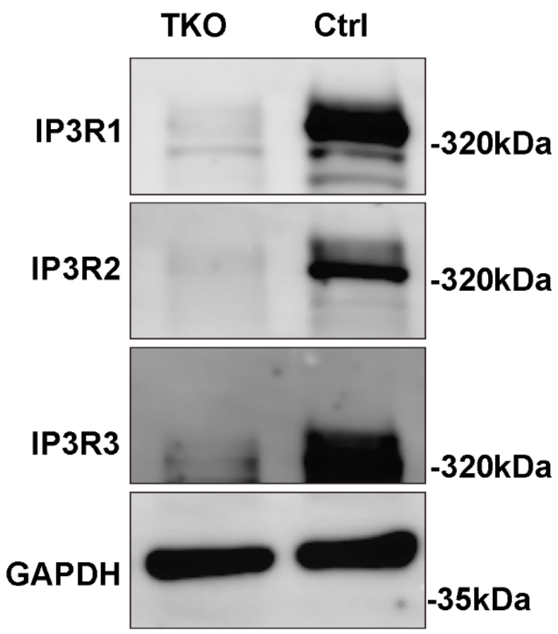
TKO
Ctrl
IP3R1
-320kDa
IP3R2
-320kDa
IP3R3
-320kDa
GAPDH
-35kDa

Source data for Fig. 2E

Original image files from which Fg. 2E was assembled.

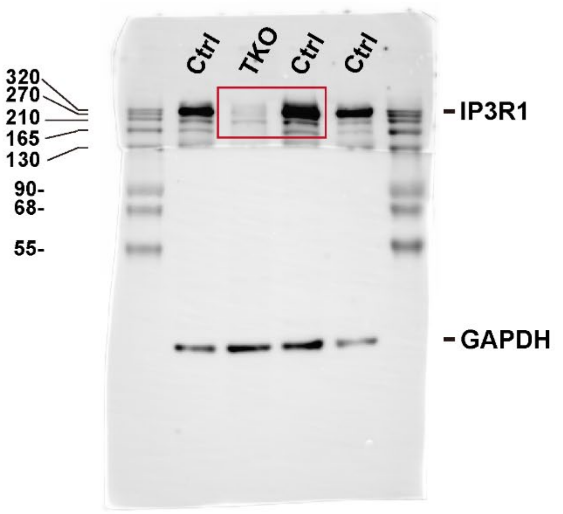

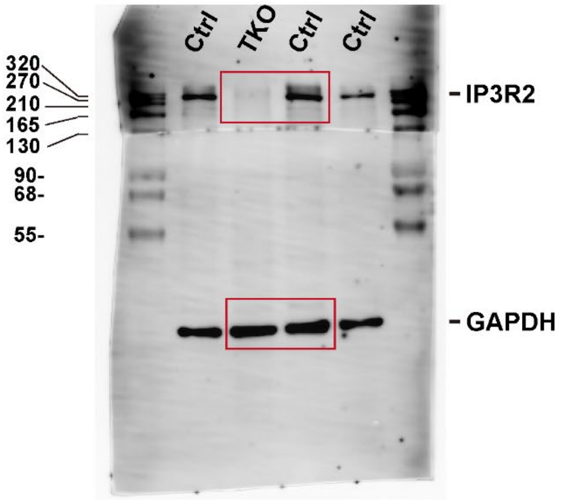

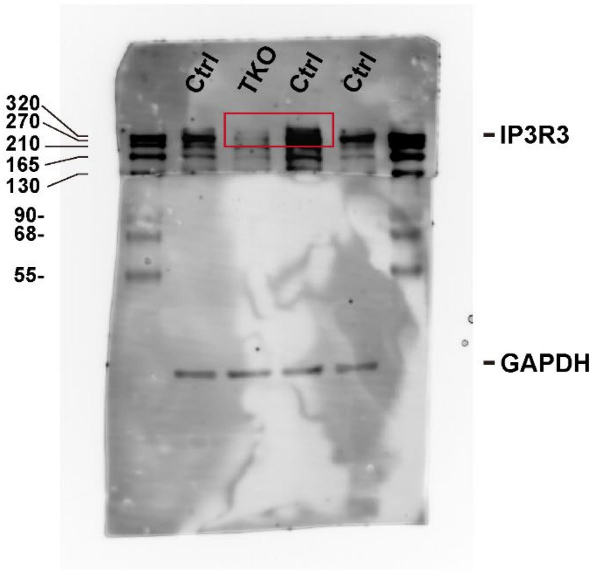

Supplement: SourceData F2 — is the source file for Fig. 2. [file jcb_202506032_sourcedataf2.pdf]
